# Association of plasma growth differentiation factor 15 with wake after sleep onset in mild cognitive impairment

**DOI:** 10.1016/j.jnha.2026.100991

**Published:** 2026-09-23

**Authors:** Teruaki Masuda, Takuya Ataka, Kaori Sumi, Yosuke Takeuchi, Yuko Akiyoshi, Fusa Matsuzaki, Hirofumi Hirakawa, Etsuro Matsubara, Noriyuki Kimura

**Affiliations:** a Department of Neurology, Faculty of Medicine, Oita University, Oita, Japan; b Department of Neuropsychiatry, Faculty of Medicine, Oita University, Oita, Japan

**Keywords:** Growth differentiation factor 15, Wake after sleep onset, Mild cognitive impairment, Wearable device, Sleep continuity

## Abstract

**Objectives:**

Circulating growth differentiation factor 15 (GDF-15) concentrations increase with age and are elevated in chronic diseases, but evidence regarding habitual wake after sleep onset (WASO) and circulating GDF-15 remains limited. We therefore examined the cross-sectional association between plasma GDF-15 and wearable-estimated WASO in older adults with mild cognitive impairment (MCI).

**Design:**

Cross-sectional biomarker analysis of first-year data from a prospective community-based cohort.

**Setting:**

USUKI study, Usuki, Oita, Japan.

**Participants:**

118 community-dwelling adults aged ≥65 years with MCI.

**Intervention:**

None.

**Measurements:**

We measured plasma GDF-15 concentrations using an enzyme-linked immunosorbent assay. We averaged wearable sleep data across repeated assessments. WASO was the primary measure of sleep continuity; total sleep time (TST) was examined as a measure of sleep quantity; and amyloid burden was assessed using ^11^C-Pittsburgh compound B PET (PiB-PET) global standardized uptake value ratio (SUVR) and PiB positivity.

**Results:**

The median plasma GDF-15 concentration was 1,230.8 pg/mL (IQR, 956.6–1,590.5), and the median WASO was 18.4 min (IQR, 11.6–27.5). After adjustment for age, sex, education, diabetes mellitus, and estimated glomerular filtration rate, each 10-min increment in WASO was associated with a 10.6% higher GDF-15 concentration (95% CI, 4.8%–16.8%; *p* < 0.001). TST and PiB-PET global SUVR were not significantly associated with GDF-15, and the proportion of PiB-positive participants did not differ across GDF-15 tertiles.

**Conclusion:**

In older adults with MCI, poorer habitual nocturnal sleep continuity, as reflected by greater wearable-estimated WASO, was associated with higher plasma GDF-15 concentrations after multivariable adjustment.

## Highlights

• Greater WASO was associated with higher plasma GDF-15 in older adults with MCI.
• A 10-min increment in WASO corresponded to 10.6% higher plasma GDF-15.
• Adding WASO to baseline covariates increased model *R*^2^ by 6.3 percentage points.
• Total sleep time and amyloid burden showed no clear relationship with plasma GDF-15.

## 1 Introduction

Mild cognitive impairment (MCI) is a clinical syndrome characterized by objective cognitive impairment with largely preserved functional independence and an increased risk of progression to dementia [1,2]. In older adults, a systematic review and meta-analysis reported that sleep problems were associated with an increased risk of subsequent cognitive impairment and Alzheimer’s disease [3]. In a prospective cohort study using actigraphy, greater sleep fragmentation was associated with a higher risk of incident Alzheimer’s disease and a faster rate of cognitive decline [4]. Among individuals with MCI, pooled objective sleep data indicate greater wake after sleep onset (WASO), a quantitative measure of sleep continuity, and lower sleep efficiency than in cognitively unimpaired older adults [5].

Circulating growth differentiation factor 15 (GDF-15) is a stress-responsive protein associated with mitochondrial dysfunction and metabolic stress [6,7]. Its concentrations increase with age and are also elevated in inflammatory states, renal dysfunction, and diverse chronic diseases [[7], [8], [9]]. These associations indicate that GDF-15 is a nonspecific marker of systemic stress and disease burden. Despite preserved sleep duration, two nights of experimentally fragmented sleep in healthy adults reduced insulin sensitivity, shifted sympathovagal balance toward sympathetic activity, and increased morning cortisol levels, whereas markers of systemic inflammation were unchanged [10]. These metabolic, autonomic, and neuroendocrine changes provide an indirect rationale for examining whether habitual sleep continuity is associated with circulating GDF-15.

An actigraphy study of women with objective or paradoxical insomnia assessed GDF-15 together with WASO and other sleep-continuity measures [11]. In that study, GDF-15 concentrations did not differ significantly between women with insomnia and controls, but the participant-level WASO–GDF-15 association was not reported. A large study of community-dwelling older adults reported no association between device-measured sleep time and serum GDF-15 but did not examine WASO or other measures of sleep continuity [12]. Evidence regarding habitual WASO and circulating GDF-15 therefore remains limited.

In this study, we examined plasma GDF-15 in relation to wearable-estimated WASO, the primary sleep measure, in the USUKI cohort of community-dwelling older adults with MCI in Japan, in which objective sleep measures were previously associated longitudinally with brain amyloid burden [13]. To our knowledge, the WASO–GDF-15 association has not been examined in older adults with MCI. For comparison, total sleep time (TST) was examined as a measure of sleep quantity, and amyloid burden was assessed using two ^11^C-Pittsburgh compound B positron emission tomography (PiB-PET) measures: global standardized uptake value ratio (SUVR) and PiB positivity. Given the physiological changes produced by experimental sleep fragmentation [10], we hypothesized that greater WASO would be associated with higher plasma GDF-15 concentrations.

## 2 Materials and methods

### 2.1 Study design and participants

This cross-sectional biomarker analysis included 118 community-dwelling adults aged 65 years or older with MCI from the USUKI study, a prospective cohort study conducted in Usuki, Oita Prefecture, Japan. Participants were recruited between August 2015 and April 2017. Baseline plasma GDF-15 concentrations, cognitive assessments, and positron emission tomography (PET) measures were examined in relation to participant-level averages of wearable-derived measures collected repeatedly during the first study year. The cohort design and assessment procedures were described previously [13,14].

Participants were included if plasma GDF-15 measurements, valid wearable-derived sleep and physical activity data, cognitive assessments, and PET imaging data were available. MCI was diagnosed on the basis of subjective and objective memory impairment, a Clinical Dementia Rating score of 0.5, and preserved or minimally impaired activities of daily living. All participants had Hachinski ischemic scores of 4 or less. Exclusion criteria were other neurological or psychiatric disorders, severe cardiac failure, severe hepatic or renal dysfunction, severe head trauma, alcoholism, stroke, difficulty walking, and ongoing cancer treatment.

The study was approved by the Ethics Committee of Oita University Hospital (B15-006; UMIN000017442) and was conducted in accordance with the Declaration of Helsinki. All participants provided written informed consent. Reporting followed the Strengthening the Reporting of Observational Studies in Epidemiology (STROBE) statement [15].

### 2.2 Plasma GDF-15 measurement

Blood samples were collected in the morning after an overnight fast whenever possible. Samples were centrifuged at 1800 × *g* for 10 min at 4 °C, and plasma was stored at −80 °C until analysis. Plasma GDF-15 concentrations were measured in pg/mL using a commercially available enzyme-linked immunosorbent assay according to the manufacturer’s protocol (Human GDF-15 Quantikine ELISA Kit, DGD150; R&D Systems, Minneapolis, MN, USA).

### 2.3 Wearable-derived sleep and physical activity assessments

Sleep and physical activity were assessed with a wrist-worn device (Silmee W20; TDK Corporation, Tokyo, Japan), as described previously [13,14]. Participants wore the device continuously, except while bathing, for 7 consecutive days at 3-month intervals during the first study year. Valid data required at least 3 days within one measurement period and at least 2 measurement periods during the year. Non-wear periods were identified from heart-rate data and were excluded, and values were averaged across all valid measurement days for each participant. The device-derived sleep duration and walking steps were previously compared with video observation [13,14], but WASO was not validated against polysomnography.

Wearable-estimated WASO was the primary sleep measure, and TST was examined as a measure of sleep quantity. Sleep onset was defined as the start of the first nocturnal period of at least 20 min without detected movement. WASO was the summed duration of device-classified awake episodes lasting at least 5 min but less than 90 min after sleep onset; an awake episode lasting at least 90 min marked sleep end. TST was the cumulative device-derived sleep time between sleep onset and sleep end, as previously reported [13,14]. Secondary sleep measures included sleep efficiency, calculated as TST/(TST + WASO) × 100, awake time count, defined as the number of these episodes, and nap TST.

Physical activity was derived from triaxial accelerometer data summarized in 1-min epochs, from which sleep-classified epochs were excluded. Activity intensity was categorized as sedentary time (≤1.5 metabolic equivalents [METs]), light physical activity (LPA; 1.6–2.9 METs), or moderate-to-vigorous physical activity (MVPA; ≥3.0 METs). Walking steps and heart rate were also obtained from the device.

### 2.4 Cognitive and neuroimaging assessments

The Mini-Mental State Examination (MMSE), Alzheimer’s Disease Assessment Scale–Cognitive Subscale (ADAS-Cog), and Japanese version of the Montreal Cognitive Assessment (MoCA-J) were used to characterize cognitive performance within the MCI cohort.

Neuroimaging assessments included PiB-PET, ^18^F-fluorodeoxyglucose PET (FDG-PET), and structural magnetic resonance imaging (MRI). PET acquisition and image-processing procedures were described previously [13]. PiB and FDG uptake were expressed as SUVRs using the cerebellar cortex as the reference region, and global SUVRs were calculated from the frontal, temporoparietal, and posterior cingulate regions. PiB-PET global SUVR and PiB positivity were used as measures of amyloid burden; for the present analysis, PiB positivity was defined as a global SUVR of ≥1.2. Gray matter and hippocampal volumes were expressed as ratios to intracranial volume, and medial temporal atrophy was assessed using a voxel-based Z score.

### 2.5 Statistical analysis

Because plasma GDF-15 concentrations were right-skewed, ln-transformed values were used for linear regression analyses. Continuous and categorical variables were summarized as medians with interquartile ranges and n (%), respectively. Participant characteristics were compared across GDF-15 tertiles using the Kruskal–Wallis test for continuous variables and the χ^2^ or Fisher exact test for categorical variables (Table 1 and Supplementary Table S1).

**Table 1:** Participant characteristics overall and according to plasma GDF-15 tertiles.

Variable	Overall (*n* = 118)	T1 (*n* = 40)	T2 (*n* = 39)	T3 (*n* = 39)	*p* value
GDF-15, pg/mL	1,230.8 [956.6–1,590.5]	853.3 [719.7–956.6]	1,235.5 [1,157.4–1,315.5]	2,014.7 [1,618.4–2,589.0]	<0.001
*Demographics*					
Age, years	76.0 [72.0–80.0]	72.5 [67.0–76.0]	77.0 [72.5–80.0]	79.0 [76.0–83.0]	<0.001
Male sex, *n* (%)	52 (44.1)	11 (27.5)	19 (48.7)	22 (56.4)	0.027
Education, years	12.0 [9.0–12.0]	12.0 [10.0–12.0]	12.0 [9.0–12.0]	11.0 [9.0–12.0]	0.090
BMI, kg/m^2^	23.2 [21.3–24.9]	22.0 [20.5–23.9]	24.1 [22.4–24.8]	23.6 [21.4–26.2]	0.075
*Vascular risk factors*					
Hypertension, *n* (%)	67 (56.8)	19 (47.5)	24 (61.5)	24 (61.5)	0.346
Diabetes mellitus, *n* (%)	24 (20.3)	4 (10.0)	5 (12.8)	15 (38.5)	0.003
Dyslipidemia, *n* (%)	37 (31.4)	11 (27.5)	12 (30.8)	14 (35.9)	0.720
*APOE* ε4 carriers, *n* (%)	17 (14.4)	7 (17.5)	3 (7.7)	7 (17.9)	0.344
*Renal function*					
eGFR, mL/min/1.73 m^2^	65.5 [56.9–75.0]	74.6 [65.4–79.4]	65.8 [60.4–73.7]	57.1 [45.8–63.9]	<0.001
*Cognition*					
MMSE	26.0 [25.0–27.8]	26.0 [25.0–28.0]	26.0 [25.0–27.0]	26.0 [25.0–27.0]	0.957
ADAS-Cog	7.3 [4.9–10.3]	5.3 [3.5–8.1]	7.3 [5.0–10.3]	8.3 [6.7–11.0]	<0.001
MoCA-J	22.0 [19.0–25.0]	24.0 [20.8–26.2]	21.0 [18.5–24.0]	21.0 [18.5–24.0]	0.017
*Neuroimaging*					
FDG-PET global SUVR	0.9 [0.9–1.0]	1.0 [0.9–1.0]	0.9 [0.9–1.0]	0.9 [0.8–0.9]	0.002
PiB-PET global SUVR	0.9 [0.8–1.3]	0.9 [0.8–1.3]	0.9 [0.8–1.3]	1.0 [0.8–1.3]	0.401
PiB positivity, *n* (%)	37 (31.4)	11 (27.5)	11 (28.2)	15 (38.5)	0.504
*Sleep parameters (wearable device)*					
WASO, min	18.4 [11.6–27.5]	16.1 [6.7–18.7]	18.6 [13.3–26.8]	27.2 [17.4–38.8]	<0.001
Total sleep time, min	401.2 [357.0–445.5]	407.3 [373.7–452.7]	390.1 [351.4–441.4]	402.0 [354.6–446.1]	0.679
*Physical activity (wearable device)*					
LPA, min/day	15.9 [10.4–28.6]	23.3 [13.0–36.4]	15.2 [10.6–25.8]	11.8 [7.9–17.8]	<0.001

Values are median [IQR] for continuous variables and *n* (%) for categorical variables. Tertiles of plasma GDF-15 are denoted T1 (lowest) to T3 (highest). The *p* values were obtained using the Kruskal–Wallis test for continuous variables and the χ² or Fisher exact test for categorical variables. PiB positivity was defined as a global PiB-PET SUVR of ≥1.2. ADAS-Cog indicates Alzheimer’s Disease Assessment Scale–Cognitive Subscale; *APOE*, apolipoprotein E; BMI, body mass index; eGFR, estimated glomerular filtration rate; FDG-PET, ^18^F-fluorodeoxyglucose positron emission tomography; GDF-15, growth differentiation factor 15; IQR, interquartile range; LPA, light physical activity; MMSE, Mini-Mental State Examination; MoCA-J, Japanese version of the Montreal Cognitive Assessment; PiB-PET, ^11^C-Pittsburgh compound B positron emission tomography; SUVR, standardized uptake value ratio; WASO, wake after sleep onset.

Associations of plasma GDF-15 with clinical, cognitive, neuroimaging, sleep, and physical activity variables were assessed using Spearman correlation coefficients, with the Benjamini–Hochberg false discovery rate (FDR) correction applied across the 22 unadjusted correlations. Partial Spearman correlations were adjusted for age, sex, education, diabetes mellitus, and estimated glomerular filtration rate (eGFR) and were not calculated for variables in the adjustment set; the corresponding *p* values were nominal and were not corrected for multiple testing (Supplementary Table S2).

Hierarchical linear regression used ln-transformed plasma GDF-15 as the dependent variable. The base model included these five covariates; the primary model added WASO; and the expanded model added FDG-PET global SUVR and LPA (Table 2; complete models in Supplementary Table S3, Panel A). As a secondary analysis, the expanded model assessed whether the WASO association persisted after additional adjustment for FDG-PET global SUVR, a measure of global cerebral glucose metabolism, and LPA, a device-derived measure of light-intensity daytime physical activity. Neither variable was interpreted as a confounder or mediator. The percentage difference in plasma GDF-15 per 10-min increment in WASO was calculated as [exp(10 × *B*) − 1] × 100.

**Table 2:** Hierarchical multivariable linear regression models of natural log-transformed plasma GDF-15.

Variable	Base model			Primary model			Expanded model		
	*B* (95% CI)	β	*p* value	*B* (95% CI)	β	*p* value	*B* (95% CI)	β	*p* value
Age, years	0.0192 (0.0056 to 0.0327)	0.227	0.006	0.0153 (0.0023 to 0.0283)	0.182	0.021	0.0097 (−0.0042 to 0.0235)	0.115	0.168
Male sex (vs female)	−0.1746 (−0.3195 to −0.0296)	−0.174	0.019	−0.0907 (−0.2350 to 0.0537)	−0.091	0.216	−0.0466 (−0.1979 to 0.1047)	−0.047	0.542
Education, years	−0.0514 (−0.0907 to −0.0121)	−0.192	0.011	−0.0436 (−0.0810 to −0.0061)	−0.162	0.023	−0.0422 (−0.0792 to −0.0051)	−0.157	0.026
Diabetes mellitus	0.2765 (0.0994 to 0.4537)	0.224	0.002	0.2596 (0.0916 to 0.4276)	0.210	0.003	0.2628 (0.0962 to 0.4294)	0.213	0.002
eGFR, mL/min/1.73 m^2^	−0.0134 (−0.0190 to −0.0078)	−0.373	<0.001	−0.0119 (−0.0173 to −0.0066)	−0.333	<0.001	−0.0120 (−0.0173 to −0.0067)	−0.334	<0.001
WASO, min	—	—	—	0.0101 (0.0047 to 0.0155)	0.278	<0.001	0.0089 (0.0034 to 0.0143)	0.244	0.002
FDG-PET global SUVR	—	—	—	—	—	—	−0.7482 (−1.7241 to 0.2278)	−0.118	0.132
LPA, min/day	—	—	—	—	—	—	−0.0031 (−0.0075 to 0.0013)	−0.102	0.162
*R*^2^	0.436			0.499			0.520		
Adjusted *R*^2^	0.411			0.472			0.485		
Δ*R*^2^ vs preceding model	—			0.063			0.021		

The dependent variable was natural log-transformed plasma GDF-15. All models included 118 participants. The base model included age, sex, education, diabetes mellitus, and eGFR. The primary model added WASO; the expanded model additionally included FDG-PET global SUVR and LPA. Em dashes indicate variables not entered in that model. *B* indicates the unstandardized regression coefficient; β, the standardized regression coefficient; CI, confidence interval; eGFR, estimated glomerular filtration rate; FDG-PET, ^18^F-fluorodeoxyglucose positron emission tomography; GDF-15, growth differentiation factor 15; LPA, light physical activity; SUVR, standardized uptake value ratio; WASO, wake after sleep onset. Sex was coded as male = 1 and female = 0. Δ*R^2^* is the increment in *R^2^* over the immediately preceding model; for the expanded model it represents the joint addition of FDG-PET global SUVR and LPA. The primary- and expanded-model WASO coefficients correspond to 10.6% and 9.3% higher plasma GDF-15 concentrations per 10-min increment, respectively. The intermediate model that added FDG-PET global SUVR to the primary model is shown in Supplementary Table S3, Panel A.

Robustness of the primary model was evaluated using heteroscedasticity-consistent type 3 (HC3) standard errors and bootstrap 95% confidence intervals from 2000 resamples. Model diagnostics are reported in Supplementary Table S3, Panel B. Sensitivity analyses replaced WASO in the primary model with sleep efficiency or awake time count (Supplementary Table S3, Panel C), and exploratory analyses of WASO, sleep efficiency, and awake time count were stratified by PiB status (Supplementary Table S3, Panel D). All tests were two-sided. Statistical significance was defined as FDR-adjusted *q* < 0.05 for the 22 unadjusted correlations and *p* < 0.05 for all other analyses. Analyses were performed using SPSS Statistics version 29.0.2.0 (IBM Corp., Armonk, NY, USA) and R version 4.3.3 (R Foundation for Statistical Computing, Vienna, Austria).

## 3 Results

### 3.1 Participant characteristics and plasma GDF-15 concentrations

A total of 118 community-dwelling older adults with MCI were included. The median age was 76.0 years [IQR, 72.0–80.0], and 52 participants (44.1%) were men. The median plasma GDF-15 concentration was 1,230.8 pg/mL [IQR, 956.6–1,590.5]. Supplementary Fig. S1 shows the raw and natural log-transformed distributions. Median wearable-estimated WASO and TST were 18.4 min [IQR, 11.6–27.5] and 401.2 min [IQR, 357.0–445.5], respectively. The median PiB-PET global SUVR was 0.9 [IQR, 0.8–1.3], and 37 participants (31.4%) were PiB positive (Table 1).

Median plasma GDF-15 concentrations were 853.3 pg/mL [IQR, 719.7–956.6] in the lowest tertile and 2,014.7 pg/mL [IQR, 1,618.4–2,589.0] in the highest tertile (Table 1). Among the primary-model covariates, higher GDF-15 tertiles were characterized by older age, higher proportions of men and participants with diabetes mellitus, and lower eGFR (all *p* ≤ 0.03). WASO increased from 16.1 min [IQR, 6.7–18.7] in the lowest tertile to 27.2 min [IQR, 17.4–38.8] in the highest tertile (*p* < 0.001), whereas TST did not differ across tertiles (*p* = 0.679). Supplementary Table S1 provides additional characteristics according to plasma GDF-15 tertiles, including MRI, secondary sleep, and other physical activity measures.

### 3.2 Association between plasma GDF-15 and wearable-estimated WASO

Plasma GDF-15 was positively correlated with WASO (Spearman’s ρ = 0.449, *p* < 0.001; FDR-adjusted *q* < 0.001; Supplementary Table S2 and Supplementary Fig. S2A). The association persisted after adjustment for age, sex, education, diabetes mellitus, and eGFR (partial ρ = 0.301, *p* < 0.001; Supplementary Table S2).

In hierarchical linear regression, the base model explained 43.6% of the variance in ln-transformed plasma GDF-15 (Table 2). Adding WASO increased the explained variance to 49.9% (Δ*R*^2^ = 0.063). In this primary model, higher WASO was associated with higher ln GDF-15 (*B* = 0.0101 per min; 95% CI, 0.0047 to 0.0155; standardized β = 0.278; *p* < 0.001). The WASO coefficient corresponded to a 10.6% higher plasma GDF-15 concentration per 10-min increment.

In the expanded model, which also included FDG-PET global SUVR and LPA, WASO remained associated with ln GDF-15 (*B* = 0.0089; 95% CI, 0.0034 to 0.0143; standardized β = 0.244; *p* = 0.002), corresponding to a 9.3% higher plasma GDF-15 concentration per 10-min increment. Neither FDG-PET global SUVR (*p* = 0.132) nor LPA (*p* = 0.162) was significantly associated with ln GDF-15, and the expanded model explained 52.0% of the variance. The complete hierarchical models are shown in Supplementary Table S3, Panel A.

Although the White test suggested possible heteroscedasticity (*p* = 0.024), the association remained statistically significant when HC3 robust standard errors were used (95% CI, 0.0028 to 0.0174; *p* = 0.007), and the bootstrap 95% CI from 2000 resamples excluded zero (0.0035 to 0.0164; Supplementary Table S3, Panel B).

### 3.3 Comparison and exploratory analyses

For comparison, neither TST nor PiB-PET global SUVR was associated with plasma GDF-15 before or after covariate adjustment (Supplementary Table S2), and the proportion of PiB-positive participants did not differ across GDF-15 tertiles (Table 1). Supplementary Figs. S2B and S2C show scatter plots for TST and PiB-PET global SUVR.

Among secondary device-derived sleep-continuity measures, lower sleep efficiency and higher awake time count were associated with higher plasma GDF-15 before and after covariate adjustment (Supplementary Table S2). In sensitivity analyses using the primary-model covariates, lower sleep efficiency (*B* = −0.0389, *p* < 0.001) and higher awake time count (*B* = 0.3671, *p* = 0.001) were associated with higher ln GDF-15 (Supplementary Table S3, Panel C). Sleep efficiency incorporates WASO, and awake time count is based on the same awake episodes used to calculate WASO; neither measure is independent of WASO.

Stratification by amyloid status showed nominal associations of WASO, sleep efficiency, and awake time count with ln GDF-15 among PiB-negative participants (*n* = 81). Among PiB-positive participants (*n* = 37), all three coefficients were in the same direction as in the PiB-negative subgroup, but none reached statistical significance; for WASO, *B* = 0.0095 (*p* = 0.066; Supplementary Table S3, Panel D).

Exploratory analyses of cognitive and neuroimaging measures are summarized in Supplementary Table S2. Among these measures, only normalized gray matter volume remained nominally associated with plasma GDF-15 after covariate adjustment (partial ρ = −0.238, *p* = 0.014); the other unadjusted associations were attenuated.

## 4 Discussion

In this cross-sectional biomarker analysis of older adults with MCI, greater wearable-estimated WASO was associated with higher plasma GDF-15 concentrations after adjustment for age, sex, education, diabetes mellitus, and eGFR. Each 10-min increment in WASO corresponded to a 10.6% higher plasma GDF-15 concentration. By contrast, no significant association was observed between plasma GDF-15 and either TST or PiB-PET global SUVR, and PiB positivity did not differ significantly across GDF-15 tertiles. These findings suggest an association of plasma GDF-15 with sleep continuity that was not detected for sleep duration.

Experimental findings provide indirect biological plausibility for the observed WASO–GDF-15 association but do not establish a mechanism linking sleep fragmentation to GDF-15. In healthy adults, experimental sleep fragmentation produced metabolic, autonomic, and neuroendocrine changes despite preserved sleep duration [10]. Separately, in experimental models, skeletal muscle mitochondrial stress induced GDF-15, which acted as a circulating regulator of systemic energy homeostasis [16]. However, GDF-15 was not measured in the sleep-fragmentation experiment [10], and sleep fragmentation was not examined in the mitochondrial-stress models [16]. TST primarily reflects sleep quantity, whereas WASO reflects sleep continuity; this distinction may help explain why plasma GDF-15 was associated with WASO but not TST. Prior human studies examined GDF-15 in relation to sleep duration [12] or compared groups defined by insomnia status [11]; the present analysis instead examined continuous, participant-level variation in wearable-estimated WASO.

Circulating GDF-15 reflects multiple aspects of systemic aging and disease: it increases with age [7], is associated with renal dysfunction [9], and has been linked to frailty-related phenotypes [[17], [18], [19]]. Consistent with this broader context, eGFR was inversely associated with GDF-15 in the primary multivariable model. Yet the WASO association persisted after multivariable adjustment and was only modestly attenuated in the secondary expanded model. Whether poorer sleep continuity contributes to higher GDF-15 concentrations or co-occurs with them cannot be determined from cross-sectional data.

In addition to the primary WASO–GDF-15 finding, exploratory cognitive and neuroimaging analyses showed no consistent pattern of association with circulating GDF-15 after covariate adjustment. Although circulating GDF-15 has been associated with poorer cognitive performance [20,21], associations with cognitive measures in the present cohort were attenuated after covariate adjustment. Concurrent amyloid burden also showed no clear relationship with plasma GDF-15, despite the longitudinal association between objective sleep measures and amyloid burden previously reported in this cohort [13]. At the disease level, circulating GDF-15 was reported to be higher in patients with type 2 diabetes but not in patients with Alzheimer’s disease relative to healthy aging [22]. The nominal association with normalized gray matter volume after covariate adjustment is broadly consistent with prior reports linking higher GDF-15 concentrations to lower brain volumes and greater white matter hyperintensity burden [23,24].

Strengths of this study include repeated wearable sleep assessments under free-living conditions across the first study year, which reduce reliance on single-night measurement. All participants in this community-based MCI cohort underwent amyloid PET assessment, allowing concurrent amyloid burden to be evaluated across the cohort. However, the cross-sectional design precludes assessment of temporal direction or causality. WASO was a movement-defined estimate without validation against polysomnography, and sleep staging was unavailable. Sleep-disordered breathing was not assessed, so respiratory-event–related sleep disruption could not be distinguished from other causes of WASO. Plasma GDF-15 was measured once, whereas sleep measures were averaged across repeated periods, so within-person covariation could not be examined. The single community-based cohort of older adults with MCI limits generalizability, and the amyloid-status subgroup analyses were exploratory, particularly given the small PiB-positive subgroup. Longitudinal studies with repeated concurrent measurements of plasma GDF-15 and device-derived sleep continuity are needed to determine whether the two measures change together over time.

In this cohort of older adults with MCI, poorer habitual nocturnal sleep continuity, as reflected by greater wearable-estimated WASO, was associated with higher plasma GDF-15 concentrations after multivariable adjustment.

## CRediT authorship contribution statement

Teruaki Masuda: Conceptualization, Formal analysis, Funding acquisition, Investigation, Methodology, Project administration, Supervision, Writing – original draft, Writing – review & editing. Takuya Ataka: Investigation, Resources, Writing – review & editing. Kaori Sumi: Resources, Writing – review & editing. Yosuke Takeuchi: Resources, Writing – review & editing. Yuko Akiyoshi: Investigation, Data curation, Writing – review & editing. Fusa Matsuzaki: Formal analysis, Data curation, Writing – review & editing. Hirofumi Hirakawa: Investigation, Writing – review & editing. Etsuro Matsubara: Funding acquisition, Resources, Writing – review & editing. Noriyuki Kimura: Funding acquisition, Resources, Supervision, Writing – review & editing.

## Declaration of Generative AI and AI-assisted technologies in the writing process

During the preparation of this work, the authors used ChatGPT (OpenAI) and Claude (Anthropic) for English language editing and for improving manuscript readability. After using these tools, the authors reviewed and edited the content as needed, and they take full responsibility for the content of the published work.

## Funding

This study was supported by JSPS KAKENHI Grant Number JP23K06911, 10.13039/100009619AMED under Grant Number 18he1402003, and 10.13039/100009619AMED under Grant Number 26dk0207080. The funding sources had no role in the design and conduct of the study; in the collection, analysis, and interpretation of data; in the preparation of the manuscript; or in the review or approval of the manuscript.

## Data availability

The data that support the findings of this study are available from the corresponding author upon reasonable request. The data are not publicly available because of privacy and ethical restrictions.

## Declaration of competing interest

The authors declare that they have no competing interests.
