# Supplementary material for: Association of plasma growth differentiation factor 15 with wake after sleep onset in mild cognitive impairment
Source: J Nutr Health Aging. 2026 Sep 23;30(11):100991. doi: 10.1016/j.jnha.2026.100991 (PMC13634698; doi:10.1016/j.jnha.2026.100991)
Supplement: Supplementary file 1 [file mmc1.docx]

**Supplementary Table S1.** Additional participant characteristics overall and according to plasma GDF-15 tertiles

| **Variable** | **Overall (*n* = 118)** | **T1 (*n* = 40)** | **T2 (*n* = 39)** | **T3 (*n* = 39)** | ***p* value** |
| --- | --- | --- | --- | --- | --- |
| *Renal function* |  |  |  |  |  |
| Creatinine, mg/dL | 0.8 [0.6–0.9] | 0.6 [0.6–0.8] | 0.8 [0.6–0.9] | 0.9 [0.8–1.0] | <0.001 |
| *Neuroimaging* |  |  |  |  |  |
| Normalized gray matter volume | 0.29 [0.27–0.32] | 0.31 [0.29–0.32] | 0.28 [0.27–0.31] | 0.28 [0.26–0.30] | 0.002 |
| Normalized hippocampal volume | 0.005 [0.004–0.005] | 0.005 [0.004–0.005] | 0.005 [0.004–0.005] | 0.004 [0.004–0.005] | 0.016 |
| Medial temporal atrophy index (VSRAD Z score) | 0.9 [0.6–1.4] | 0.8 [0.6–1.1] | 1.0 [0.6–1.6] | 1.2 [0.8–1.6] | 0.024 |
| *Sleep parameters (wearable device)* |  |  |  |  |  |
| Sleep efficiency, % | 95.6 [93.4–97.3] | 96.4 [95.7–98.2] | 95.5 [93.5–96.9] | 93.9 [90.9–96.5] | <0.001 |
| Awake time count, episodes/night | 0.5 [0.3–0.7] | 0.4 [0.2–0.5] | 0.5 [0.3–0.6] | 0.7 [0.4–1.0] | <0.001 |
| Nap TST, min | 35.5 [19.6–59.7] | 33.0 [16.7–58.3] | 35.5 [22.7–56.6] | 43.5 [23.2–67.7] | 0.539 |
| *Physical activity (wearable device)* |  |  |  |  |  |
| Walking steps/day | 4,297 [2,771–6,212] | 4,754 [3,381–7,184] | 4,100 [2,788–5,985] | 3,597 [2,069–5,809] | 0.032 |
| MVPA, min/day | 21.2 [11.2–34.0] | 24.0 [15.2–41.2] | 22.3 [10.6–33.1] | 15.4 [8.1–25.6] | 0.037 |
| Sedentary time, min/day | 786.7 [739.6–829.8] | 771.2 [745.6–805.2] | 814.4 [736.1–836.7] | 793.8 [746.4–837.9] | 0.165 |
| Heart rate, bpm | 63.9 [59.6–68.8] | 65.5 [60.6–68.8] | 61.8 [57.9–68.0] | 62.4 [59.1–69.1] | 0.446 |

Values are median [IQR]. Tertiles of plasma GDF-15 are denoted T1 (lowest) to T3 (highest). The *p* values were obtained using the Kruskal–Wallis test. Abbreviations used throughout the Supplementary Tables: ADAS-Cog, Alzheimer’s Disease Assessment Scale–Cognitive Subscale; BMI, body mass index; bpm, beats per minute; eGFR, estimated glomerular filtration rate; FDG-PET, ^18^F-fluorodeoxyglucose positron emission tomography; GDF-15, growth differentiation factor 15; IQR, interquartile range; LPA, light physical activity; MMSE, Mini-Mental State Examination; MoCA-J, Japanese version of the Montreal Cognitive Assessment; MVPA, moderate-to-vigorous physical activity; PiB-PET, ^11^C-Pittsburgh compound B positron emission tomography; SUVR, standardized uptake value ratio; TST, total sleep time; VSRAD, Voxel-based Specific Regional Analysis System for Alzheimer’s disease; WASO, wake after sleep onset. Gray matter and hippocampal volumes were expressed as ratios to intracranial volume. Gray matter and hippocampal volume data were available for 107 participants, and VSRAD Z scores were available for 109 participants; analyses were based on available cases.

**Supplementary Table S2.** Correlations of plasma GDF-15 with clinical, cognitive, neuroimaging, sleep, and physical activity variables

| **Variable** | ***n*** | **Spearman’s ρ** | ***p* value** | **FDR-adjusted *q*** | **Partial ρ** | **Partial *p* value** |
| --- | --- | --- | --- | --- | --- | --- |
| *Demographics* |  |  |  |  |  |  |
| Age, years | 118 | 0.465 | <0.001 | <0.001 | — | — |
| Education, years | 118 | −0.211 | 0.022 | 0.030 | — | — |
| BMI, kg/m^2^ | 118 | 0.148 | 0.109 | 0.142 | 0.007 | 0.942 |
| *Renal function* |  |  |  |  |  |  |
| eGFR, mL/min/1.73 m^2^ | 118 | −0.486 | <0.001 | <0.001 | — | — |
| Creatinine, mg/dL | 118 | 0.513 | <0.001 | <0.001 | 0.205 | 0.026 |
| *Cognition* |  |  |  |  |  |  |
| MMSE | 118 | −0.050 | 0.594 | 0.623 | 0.135 | 0.144 |
| ADAS-Cog | 118 | 0.370 | <0.001 | <0.001 | 0.083 | 0.371 |
| MoCA-J | 118 | −0.264 | 0.004 | 0.006 | −0.016 | 0.867 |
| *Neuroimaging* |  |  |  |  |  |  |
| PiB-PET global SUVR | 118 | 0.081 | 0.385 | 0.423 | 0.013 | 0.888 |
| FDG-PET global SUVR | 118 | −0.336 | <0.001 | <0.001 | −0.155 | 0.093 |
| Normalized gray matter volume | 107 | −0.384 | <0.001 | <0.001 | −0.238 | 0.014 |
| Normalized hippocampal volume | 107 | −0.296 | 0.002 | 0.004 | −0.011 | 0.911 |
| Medial temporal atrophy index (VSRAD Z score) | 109 | 0.289 | 0.002 | 0.004 | 0.016 | 0.869 |
| *Sleep parameters (wearable device)* |  |  |  |  |  |  |
| WASO, min | 118 | 0.449 | <0.001 | <0.001 | 0.301 | <0.001 |
| Total sleep time, min | 118 | 0.027 | 0.775 | 0.775 | −0.044 | 0.635 |
| Sleep efficiency, % | 118 | −0.416 | <0.001 | <0.001 | −0.291 | 0.001 |
| Awake time count, episodes/night | 118 | 0.449 | <0.001 | <0.001 | 0.270 | 0.003 |
| Nap TST, min | 118 | 0.093 | 0.316 | 0.366 | 0.001 | 0.991 |
| *Physical activity (wearable device)* |  |  |  |  |  |  |
| Walking steps/day | 118 | −0.270 | 0.003 | 0.005 | −0.140 | 0.131 |
| Sedentary time, min/day | 118 | 0.106 | 0.255 | 0.311 | 0.104 | 0.262 |
| LPA, min/day | 118 | −0.351 | <0.001 | <0.001 | −0.142 | 0.125 |
| MVPA, min/day | 118 | −0.245 | 0.008 | 0.011 | −0.177 | 0.055 |

Partial Spearman correlations were adjusted for age, sex, education, diabetes mellitus, and eGFR; partial correlations were not calculated for variables included in the adjustment set (em dashes). FDR-adjusted *q* values were calculated using the Benjamini–Hochberg method across all 22 variables. FDR indicates false discovery rate. Other abbreviations are as defined in Supplementary Table S1.

**Supplementary Table S3.** Additional regression, robustness, and sensitivity analyses

***Panel A.*** *Complete hierarchical linear regression models*

| **Variable** | ***B*** | **SE** | **95% CI** | **β** | ***t*** | ***p* value** | **VIF** |
| --- | --- | --- | --- | --- | --- | --- | --- |
| *Base model (n = 118, R^2^ = 0.436, adjusted R^2^ = 0.411, F = 17.33, p < 0.001)* | | | | | | | |
| Age, years | 0.0192 | 0.0068 | 0.0056 to 0.0327 | 0.227 | 2.801 | 0.006 | 1.30 |
| Male sex (vs female) | −0.1746 | 0.0732 | −0.3195 to −0.0296 | −0.174 | −2.386 | 0.019 | 1.06 |
| Education, years | −0.0514 | 0.0198 | −0.0907 to −0.0121 | −0.192 | −2.590 | 0.011 | 1.09 |
| Diabetes mellitus | 0.2765 | 0.0894 | 0.0994 to 0.4537 | 0.224 | 3.093 | 0.002 | 1.04 |
| eGFR, mL/min/1.73 m^2^ | −0.0134 | 0.0028 | −0.0190 to −0.0078 | −0.373 | −4.730 | <0.001 | 1.23 |
| *Primary model: base model + WASO (n = 118, R^2^ = 0.499, adjusted R^2^ = 0.472, F = 18.44, p < 0.001)* | | | | | | | |
| Age, years | 0.0153 | 0.0066 | 0.0023 to 0.0283 | 0.182 | 2.337 | 0.021 | 1.34 |
| Male sex (vs female) | −0.0907 | 0.0728 | −0.2350 to 0.0537 | −0.091 | −1.245 | 0.216 | 1.17 |
| Education, years | −0.0436 | 0.0189 | −0.0810 to −0.0061 | −0.162 | −2.306 | 0.023 | 1.10 |
| Diabetes mellitus | 0.2596 | 0.0848 | 0.0916 to 0.4276 | 0.210 | 3.063 | 0.003 | 1.04 |
| eGFR, mL/min/1.73 m^2^ | −0.0119 | 0.0027 | −0.0173 to −0.0066 | −0.333 | −4.413 | <0.001 | 1.26 |
| WASO, min | 0.0101 | 0.0027 | 0.0047 to 0.0155 | 0.278 | 3.734 | <0.001 | 1.23 |
| *Primary model + FDG-PET global SUVR (n = 118, R^2^ = 0.511, adjusted R^2^ = 0.480, F = 16.44, p < 0.001)* | | | | | | | |
| Age, years | 0.0122 | 0.0068 | −0.0013 to 0.0256 | 0.144 | 1.790 | 0.076 | 1.45 |
| Male sex (vs female) | −0.0484 | 0.0767 | −0.2004 to 0.1035 | −0.048 | −0.632 | 0.529 | 1.32 |
| Education, years | −0.0422 | 0.0188 | −0.0794 to −0.0050 | −0.157 | −2.249 | 0.027 | 1.10 |
| Diabetes mellitus | 0.2692 | 0.0843 | 0.1021 to 0.4363 | 0.218 | 3.192 | 0.002 | 1.05 |
| eGFR, mL/min/1.73 m^2^ | −0.0120 | 0.0027 | −0.0173 to −0.0067 | −0.334 | −4.470 | <0.001 | 1.26 |
| WASO, min | 0.0094 | 0.0027 | 0.0040 to 0.0147 | 0.258 | 3.441 | <0.001 | 1.26 |
| FDG-PET global SUVR | −0.8122 | 0.4925 | −1.7883 to 0.1638 | −0.128 | −1.649 | 0.102 | 1.36 |
| *Expanded model: primary model + FDG-PET global SUVR + LPA (n = 118, R^2^ = 0.520, adjusted R^2^ = 0.485, F = 14.76, p < 0.001)* | | | | | | | |
| Age, years | 0.0097 | 0.0070 | −0.0042 to 0.0235 | 0.115 | 1.387 | 0.168 | 1.55 |
| Male sex (vs female) | −0.0466 | 0.0763 | −0.1979 to 0.1047 | −0.047 | −0.611 | 0.542 | 1.32 |
| Education, years | −0.0422 | 0.0187 | −0.0792 to −0.0051 | −0.157 | −2.258 | 0.026 | 1.10 |
| Diabetes mellitus | 0.2628 | 0.0841 | 0.0962 to 0.4294 | 0.213 | 3.126 | 0.002 | 1.05 |
| eGFR, mL/min/1.73 m^2^ | −0.0120 | 0.0027 | −0.0173 to −0.0067 | −0.334 | −4.482 | <0.001 | 1.26 |
| WASO, min | 0.0089 | 0.0027 | 0.0034 to 0.0143 | 0.244 | 3.245 | 0.002 | 1.28 |
| FDG-PET global SUVR | −0.7482 | 0.4924 | −1.7241 to 0.2278 | −0.118 | −1.519 | 0.132 | 1.37 |
| LPA, min/day | −0.0031 | 0.0022 | −0.0075 to 0.0013 | −0.102 | −1.409 | 0.162 | 1.20 |

The dependent variable was natural log-transformed plasma GDF-15. Each sequential model retained the covariates from the previous model. The base model, primary model, and expanded model correspond to the models presented in Table 2; the intermediate model that added FDG-PET global SUVR to the primary model is shown here only. *B* indicates the unstandardized regression coefficient; β, the standardized regression coefficient; CI, confidence interval; SE, standard error; VIF, variance inflation factor. Sex was coded as male = 1 and female = 0. Other abbreviations are as defined in Supplementary Table S1.

***Panel B.*** *Model diagnostics and robustness checks for the primary model*

| **Domain** | **Check** | **Statistic** | ***p* value** |
| --- | --- | --- | --- |
| Residual normality | Shapiro–Wilk *W* | 0.980 | 0.071 |
| Heteroscedasticity | Breusch–Pagan test | — | 0.614 |
| Heteroscedasticity | White test | — | 0.024 |
| Multicollinearity | Maximum VIF | 1.34 | — |
| Influence | Maximum Cook’s distance | 0.21 | — |
| Influence | Participants with Cook’s distance > 4/*n* | 11 | — |
| Outliers | Maximum \|studentized residual\| | 3.716 | — |
| Robustness (HC3) | WASO coefficient | *B* = 0.0101; 95% CI, 0.0028 to 0.0174 | 0.007 |
| Robustness (bootstrap) | WASO coefficient (2,000 resamples) | 95% CI, 0.0035 to 0.0164 | — |

Diagnostics and robustness checks refer to the primary model, which included age, sex, education, diabetes mellitus, eGFR, and WASO, with natural log-transformed plasma GDF-15 as the dependent variable. HC3 indicates heteroscedasticity-consistent type 3. Other abbreviations are defined in Supplementary Table S1 and Panel A of Supplementary Table S3.

***Panel C.*** *Sensitivity analyses using alternative sleep-continuity measures*

| **Model** | **Sleep variable** | ***B*** | **95% CI** | **β** | ***p* value** | ***R*^2^** | **Δ*R*^2^ vs base** |
| --- | --- | --- | --- | --- | --- | --- | --- |
| Primary model | WASO, min | 0.0101 | 0.0047 to 0.0155 | 0.278 | <0.001 | 0.499 | 0.063 |
| Sleep efficiency model | Sleep efficiency, % | −0.0389 | −0.0616 to −0.0162 | −0.251 | <0.001 | 0.489 | 0.053 |
| Awake time count model | Awake time count, episodes/night | 0.3671 | 0.1461 to 0.5882 | 0.255 | 0.001 | 0.486 | 0.050 |

Each model included age, sex, education, diabetes mellitus, and eGFR plus one sleep-continuity measure; the primary model is shown for reference. Sleep efficiency was calculated as TST/(TST + WASO) × 100. Awake time count was based on the same nocturnal awakening episodes used to calculate WASO. The dependent variable was natural log-transformed plasma GDF-15. Δ*R*^2^ is the increment over the covariate-only base model (*R*^2^ = 0.436). Abbreviations are defined in Supplementary Table S1 and Panel A of Supplementary Table S3.

***Panel D.*** *PiB status–stratified analyses*

| **Sleep variable** | ***B*** | **95% CI** | **β** | ***p* value** | ***R*^2^** |
| --- | --- | --- | --- | --- | --- |
| PiB-negative (*n* = 81) |  |  |  |  |  |
| WASO, min | 0.0085 | 0.0012 to 0.0159 | 0.220 | 0.023 | 0.552 |
| Sleep efficiency, % | −0.0318 | −0.0620 to −0.0017 | −0.191 | 0.038 | 0.547 |
| Awake time count, episodes/night | 0.3061 | 0.0171 to 0.5951 | 0.211 | 0.038 | 0.547 |
| PiB-positive (*n* = 37) |  |  |  |  |  |
| WASO, min | 0.0095 | −0.0007 to 0.0197 | 0.283 | 0.066 | 0.484 |
| Sleep efficiency, % | −0.0360 | −0.0795 to 0.0076 | −0.254 | 0.102 | 0.471 |
| Awake time count, episodes/night | 0.3561 | −0.0810 to 0.7931 | 0.249 | 0.107 | 0.470 |

Each row represents a subgroup-specific model with natural log-transformed plasma GDF-15 as the dependent variable, adjusted for age, sex, education, diabetes mellitus, and eGFR. PiB positivity was defined as a global PiB-PET SUVR of ≥1.2. PiB indicates Pittsburgh compound B. Other abbreviations are defined in Supplementary Table S1 and Panel A of Supplementary Table S3.
