# Supplementary material for: Association of plasma growth differentiation factor 15 with wake after sleep onset in mild cognitive impairment
Source: J Nutr Health Aging. 2026 Sep 23;30(11):100991. doi: 10.1016/j.jnha.2026.100991 (PMC13634698; doi:10.1016/j.jnha.2026.100991)
Supplement: Supplementary file 2 [file mmc2.docx]

**Supplementary Figure S1.** Distribution of plasma GDF-15 concentrations.

Histograms show (A) raw and (B) natural log-transformed plasma GDF-15 concentrations among 118 participants. GDF-15 indicates growth differentiation factor 15.

**Supplementary Figure S2.** Associations of plasma GDF-15 with sleep measures and PiB-PET global SUVR.

Scatter plots show plasma GDF-15 concentrations in relation to (A) wake after sleep onset (WASO), (B) total sleep time (TST), and (C) PiB-PET global standardized uptake value ratio (SUVR). The *y*-axis is logarithmic. Spearman correlation coefficients and *p* values are shown in each panel and correspond to Supplementary Table S2. GDF-15 indicates growth differentiation factor 15; PiB-PET, ^11^C-Pittsburgh compound B positron emission tomography.
